# Factor associated with progression to chronic arterial hypertension in women with preeclampsia in Yaoundé, Cameroon

**DOI:** 10.11604/pamj.2019.33.200.16857

**Published:** 2019-07-15

**Authors:** Sylvie Ndongo Amougou, Simon Maginot Mintya’a Mbita, Dieudonne Danwe, Pierre-Marie Tebeu

**Affiliations:** 1 Department of Internal Medicine and Specialties, Faculty of Medicine and Biomedical Sciences, University of Yaounde I, Yaounde, Cameroon; 2 University Teaching Hospital of Yaounde, Yaounde, Cameroon; 3 Department of Gynaecology and Obstetrics, Faculty of Medicine and Biomedical Sciences, University of Yaounde I, Yaounde, Cameroon

**Keywords:** Associated factors, chronic hypertension, preeclampsia, Yaoundé, Cameroon

## Abstract

**Introduction:**

Hypertensive diseases in pregnancy are the leading medical problem during pregnancy. Some of the women affected remain hypertensive after pregnancy and the post-partum period. This study aimed to assess the factors associated to the persistence of hypertension after preeclampsia.

**Methods:**

This was a retrospective cohort study which included all women who had preeclampsia. The minimal follow-up period was 12 months. We excluded from the study all women who had superimposed preeclampsia. Sociodemographic data and past history were recorded and a physical exam was performed for all participants. Multivariate logistic regression was used to determine factors independently associated to the persistence of hypertension.

**Results:**

Our cohort consisted of 136 women. The mean follow-up period was 3.7 years. Thirty two women (23.53%) remained hypertensive. This represented an incidence rate of 2.85% per year. Old age (≥ 40 years), housewife occupation, multigravidity (> 4), onset of preeclampsia before 34 weeks' gestation, obesity and the presence of hypertension in siblings were factors independently associated to persistent hypertension.

**Conclusion:**

Many women affected by preeclampsia remain hypertensive after pregnancy. It is important to provide adequate follow-up for this patients in order to intervene on the factors leading to this outcome.

## Introduction

Hypertension is defined by the World Health Organization (WHO) as a systolic blood pressure more or equal to 140 mmHg and/or a diastolic blood pressure more or equal to 90 mmHg [1]. It complicates about 10% of all pregnancies and represent the first medical problem according to the American College of Obstetricians and Gynecologists. Hypertensive diseases in pregnancy are divided in 4 categories: chronic hypertension, gestational hypertension, preeclampsia (PE) and chronic hypertension with superimposed PE [2]. Chronic hypertension is defined as hypertension that is diagnosed before pregnancy or before 20 weeks of gestation. Hypertension that is first diagnosed after 20 weeks' gestation and persists for greater than 12 weeks postpartum is also considered chronic hypertension. Gestational hypertension is defined as hypertension that develops in pregnancy after 20 weeks' gestation and resolves before 12 weeks postpartum in the absence of proteinuria (<300mg of protein in 24 h). PE is a syndrome and is typically characterized as new-onset hypertension and proteinuria (>300mg of protein in 24 h) diagnosed in pregnancy often after 20 weeks' gestation [3]. However, according to the latest definitions, other findings such as new-onset thrombocytopenia, renal impairment, neurological complications, liver involvement and fetal growth restriction may substitute to new-onset proteinuria. PE can be classified either as early-onset (before 34 weeks' gestation) or late-onset (at or after 34 weeks' gestation) [4]. Hypertensive diseases in pregnancy are one of the three leading causes of maternal death in the world together with hemorrhage and sepsis/infection. They cause approximately 14% of all maternal deaths worldwide and are more frequent in low and middle income countries where their incidence keeps rising [5]. Thus, in Cameroon Tebeu *et al* found 17.5% of maternal deaths related to hypertensive diseases in pregnancy in 2007 while Foumane *et al* found 22.4% in 2010 [6, 7]. These hypertensive diseases in pregnancy, especially PE, make the women affected to be more vulnerable to future cardiovascular disease. After a PE, there may be metabolic alterations causing endothelial dysfunction, sympathetic hyperactivity, peripheral vascular and renovascular resistance, insulin resistance, hyperlipidemia and obesity. All these can in turn lead to often silent diseases such as diabetes, kidney injury and hypertension [8]. The latter is the main risk factor of stroke and coronary artery disease, which are currently the leading causes of death in the World [9]. Many studies showed that women who had PE are at least twice as likely to have ischemic heart disease and stroke compared to those who had normotensive pregnancies and this risks seem to be mediated by a greater risk of future chronic hypertension after PE [4, 10-12]. It therefore appeared necessary to us to identify the factors associated to the occurrence of chronic hypertension after PE on which we may act in order to reduce this future cardiovascular risk in our context where two thirds of cardiovascular disease mortality is registered.

## Methods

**Type of study:** we carried out a retrospective cohort study over an 8-year period from January 1^st^ 2009 to January 1^st^ 2016 in the obstetrics and gynecology department of the Yaoundé University Teaching Hospital.

**Study population:** all women who had PE during the study period were included in the study. The minimal follow-up period after pregnancy was 12 months. We excluded all women who had superimposed PE from the study.

**Data collection:** sociodemographic data and past medical history were collected from patients' medical records. We performed a physical examination of each participant including the measurement of weight, height and that of blood pressure with an automated blood pressure machine. The body mass index (BMI) was calculated from weight and height according to the formula BMI = WEIGHT/(HEIGHT)^2^.

**Statistical analysis:** we used the software SPSS version 20.0. Student's t-test was used for comparison of means. The Chi-square test and multivariable logistic regression were used to measure the association between categorical variables. A p-value less than 0.05 was considered statistically significant.

**Ethical considerations:** the study was approved by the Institutional Ethics Committee of the Faculty of Medicine and Biomedical Sciences of the University of Yaoundé I. A research authorization was obtained from the administration of the Yaoundé University Teaching Hospital and each participant gave informed consent before being included in the study.

## Results

**Figure 1:**
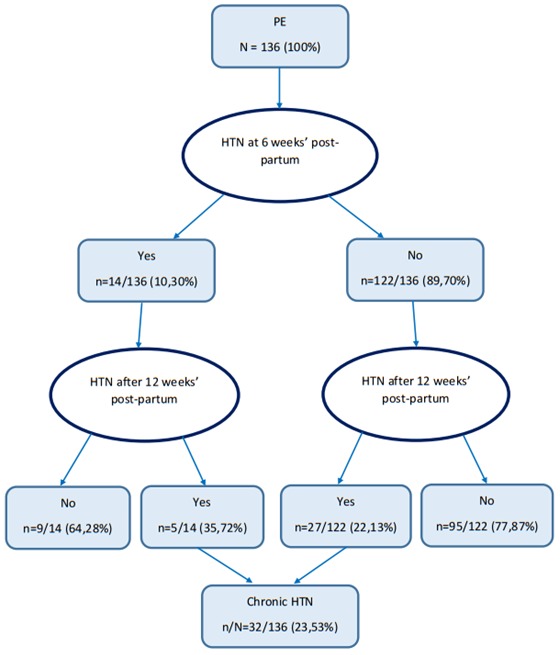
Evolution of blood pressure after pregnancy

Our cohort included 140 women who had preeclampsia. Four of them were excluded from the study because they had superimposed preeclampsia. The mean follow-up period was 3.7 years. Thirty two women had persistent hypertension at the end of our study, which gave us a prevalence of 23.53% and an incidence rate of 2.85% per year. Table 1 show the baseline characteristics of our study population. We can see that the mean age, weight, BMI and gravidity were significantly greater for women who remains hypertensive. On the other hand, the onset of PE was significantly earlier in these last. Figure 1 shows blood pressures evolution after delivery. We see that of the thirty two women who remained hypertensive, five had persistent hypertension since delivery while the other first of all had a normalization of their blood pressure which later on raised up. After adjustment for confounders using multiple logistic regression (Table 2), the factors found to be associated to persistent hypertension were: age ≥ 40 years (OR = 20.7 (1.1 - 390.0); p = 0.043), housewife profession (OR = 21,.8 (3,4- 138.3); p = 0.001), gravidity > 4 (OR = 7.9 (1.0 - 59.1); p = 0.044), onset of PE before 34 weeks' gestation (OR = 9.3 (2.1 ' 42.0); p = 0.004), presence of hypertension in siblings (OR = 6.7 (1.0 - 44.2); p = 0.047) and obesity (OR = 16.5 (2.3 - 120.6); p = 0.006).

**Table 1:** Baseline characteristics of the study population

	NormotensiveM ± σ	HypertensiveM ± σ	p
N	104	32	
Age (years)	31 ± 7	37 ± 6	0.000
Weight (kg)	67,34 ± 8,98	73,04 ± 10,39	0.003
Height (cm)	161 ± 4	160 ± 4	0.111
BMI (kg/m^2^)	25,93 ± 3,19	28,78 ± 4,03	0.000
HR (bpm)	77 ± 5	76 ± 6	0.409
Gravidity	3 ± 2	4 ± 2	0.000
Gestational age (weeks)	36 ± 3	33 ± 3	0.000

BMI: body mass index HR: heart rate M±σ: mean ± standard deviation

**Table 2:** Factors independently associated to progression to chronic hypertension

	Normotensiven (%)	Hypertensiven (%)	ORa (95% CI)	Pa
N (%)	104 (100)	32 (100)		
**Age (years)**				
≥ 40	11 (10,6)	8 (25,0)	20,7 (1,1 – 390,0)	0.043
< 40	93 (89,4)	24 (75,0)		
**Profession**				
Housewife	14 (13,5)	14 (43,8)	21,8 (3,4 – 138,3)	0,001
Others	90 (86,5)	18 (56,2)		
**HTN in siblings**				
Yes	5 (4,8)	9 (28,1)	6,7 (1,0 – 44,2)	0,047
No	99 (93,3)	23 (71,9)		
**Gestational age**				
< 34	25 (24,0)	20 (62,5)	9,3 (2,1 – 42,0)	0,004
≥ 34	79 (76,0)	12 (37,5)		
**Gravidity**				
> 4	12 (11,5)	17 (53,1)	7,9 (1,0 – 59,1)	0,044
≤ 4	92 (88,5)	15 (46,9)		
**Obesity**				
Yes	7 (6,7)	13 (40,6)	16,5 (2,3 – 120,6)	0,006
No	97 (93,3)	19 (59,4)		

N: number %: percentage HTN: hypertension OR: odds ratio

## Discussion

The aim of our study was to determine the prevalence, incidence and factors associated to the progression of PE to chronic hypertension. Thirty two women who had PE remained hypertensive at follow-up, giving us a prevalence of 23.5% in our study population. This finding is higher than the 14.8% found by Sibai *et al* in the United States of America and the 15.5% found by Festa *et al*. in Italy [13, 14]. This differences can be explained firstly by the differences in our study populations. Sibai *et al* included only primigravid women their study, but we know that the risk of PE and subsequent risk of chronic hypertension increases with parity especially when the previous pregnancies where preeclamptic. Festa *et al*. on their side included all women who had hypertensive disorders in pregnancy (gestational hypertension, chronic hypertension and preeclampsia). This may have reduce the prevalence of chronic hypertension after pregnancy which is mainly secondary to PE alone. Secondly, our study was done on an entirely black population which is more likely to develop hypertensive diseases in pregnancy and chronic hypertension. In this study housewife occupation was found to be independently associated to the progression to chronic hypertension. These women had significantly lower education levels and income (p< 0.05) and therefore low socioeconomic status. Silva *et al*. concluded in 2008 on the Generation R study that low maternal socioeconomic status is a strong risk factor for PE and that only a small part of this association can be explained by the mediating effects of established risk factors for PE [15]. So, higher risk of PE also leads to higher risk of chronic hypertension after pregnancy.

We found an association between the number of pregnancies (>4) and the risk of chronic hypertension. This can be explained by the fact that women who had a preeclamptic pregnancy are at higher risk of having PE in subsequent pregnancies. In addition in our study, women who had more than 4 pregnancies were more housewives (p<0.05). Low socioeconomic study may therefore be a mediating factor in this association. Women who had early onset PE had a 9 fold increased risk of progression to chronic hypertension. This result is consistent with those of Sibai *et al*. in the United States of America and Hwang et al in South Korea who also found early onset of PE as a risk factor of chronic hypertension [13, 16]. We assessed the link between family history of hypertension and the risk of future chronic hypertension after PE and found that the presence of hypertension in the siblings of the affected woman was independently associated to progression to chronic hypertension. The presence of a family history of hypertension especially in first degree relative is a well-known risk factor of chronic hypertension and once more points the fact that a genetic component is implicated in the pathogenesis of hypertensive diseases in pregnancy and primary hypertension. We also found that obese women who had PE were more prone to chronic hypertension. This result corroborates those of Hwang *et al*. in South Korea, Timpka *et al*. in the United States of America and Festa *et al*. in Italy who reached the same conclusion [14, 16, 17]. It can be justified firstly by the fact that obesity is an independent risk factor of PE and secondly by the fact that obesity is also associated to the risk of chronic hypertension. This results highlights the necessity to create strategies to specifically follow-up and take care of women who had PE and who have these risk factors of progression to chronic hypertension.

## Conclusion

The occurrence of chronic hypertension following PE is common in Cameroon. Several clinical and sociodemographic and economic factors are linked to this risk namely age, high gravidity, obesity, early onset PE, history of hypertension in siblings and housewife occupation. It seem therefore important to us to put in place prevention strategies for affected women.

### What is known about this topic

- Women who had preeclampsia have increased risk of progression to chronic hypertension and cardiovascular disease after pregnancy.

### What this study adds

- In Cameroonian women who had preeclampsia, old age, housewife occupation, history of hypertension in siblings, early onset PE, high gravidity and obesity are independent factor linked to the risk of progression to chronic hypertension.

## Competing interests

The authors declare no competing interests.
